# Inpatient falls in older adults: a cohort study of antihypertensive prescribing pre- and post-fall

**DOI:** 10.1186/s12877-018-0749-8

**Published:** 2018-02-23

**Authors:** H. M. R. B. Omer, J. Hodson, S. K. Pontefract, U. Martin

**Affiliations:** 1 University Hospitals Coventry and Warwickshire NHS Trust, Coventry, UK; 2 University Hospitals Birmingham NHS Trust, Birmingham, UK; 3 College of Medical and Dental Sciences, University of Birmingham, Birmingham, B15 2TT UK

**Keywords:** Antihypertensive, Medication review, Postural hypotension

## Abstract

**Background:**

Falls are common during hospital admissions and may occur more frequently in patients who are taking antihypertensive medications, particularly in the context of normal to low blood pressure. The review and adjustment of these medications is an essential aspect of the post-fall assessment and should take place as soon as possible after the fall.

Our aim was to investigate whether appropriate post-fall adjustments of antihypertensive medications are routinely made in a large National Health Service (NHS) Trust.

**Methods:**

Inpatient records over an eight-month period were captured from an electronic prescribing system to identify older adults (≥80 years old) with normal/low blood pressures (< 140 mmHg systolic) who had a documented inpatient fall as these patients were considered to be at high risk of further falls. Prescribed antihypertensive medication on admission was then compared with the post-fall (within 24 h after the fall) and discharge prescriptions.

**Results:**

A total of 146 patients were included in the analysis. Of those, 120 patients (82%) were taking the same number of antihypertensive medications in the 24 h after the fall as they were before; only 19 patients (13%) had a reduction in the number of medications and seven patients (5%) had an increase in medications during that period. Only 9% of the antihypertensive classes assessed were either stopped or reduced in dose immediately post-fall. In addition, 11 new antihypertensives were prescribed at this time.

At discharge, half of the patients (*n* = 73) remained on the same number of antihypertensive medication as on admission, 51 patients (35%) were on fewer antihypertensives and 22 (15%) were on more. Additionally, no changes were made to individual antihypertensives in 49% of prescriptions; 34% were stopped or reduced in dose but 38 new agents were started by the time of discharge. Angiotensin converting enzyme inhibitors and angiotensin II receptor blockers (ACEi/ARB) were the class of medications most commonly stopped or reduced (51%).

**Conclusions:**

Antihypertensive prescriptions are frequently unchanged after an inpatient fall. Routine medication review needs to be part of post-fall assessments in hospital to reduce the risk of further falls.

## Background

Falls are a frequent occurrence in the hospital setting, and remain the most common patient safety incident reported by organisations [1]. They have the potential to cause significant injuries, particularly among older adults, thereby extending length of stay and prolonging recovery times [2]. Falls therefore incur a substantial cost to National Health Service (NHS) hospitals [3]. With an average fall rate of 6.63 per 1000 occupied bed days across England and Wales in 2015 [1], there is an urgent need to implement preventative strategies and after care for patients post-fall.

There are multiple risk factors associated with falls, which can be broadly divided into extrinsic (due to environmental factors) and intrinsic (due to the physiology of ageing and individual comorbidities) [4]. Extrinsic factors are the commonest cause of falls in the older adult population and include physical obstacles, poor lighting, slippery upholstery and the absence of physical aids such as hand rails [5, 6]. Intrinsic factors include changes to vision, hearing, muscle power, balance and gait [4]. Physiological changes, combined with other extrinsic factors, contribute to a large proportion of falls in older adults [4, 5]. There are certain conditions which also increase the risk of falls. One such condition is orthostatic hypotension, defined as a postural drop in blood pressure of at least 20 mmHg systolic and/or 10mmgHg diastolic within 3 min of being upright [7]. During the ageing process, there is often a reduction in blood vessel compliance, which leads to an increase in systemic vascular resistance and a resultant increase in blood pressure [8]. In certain older adults however, the development of conditions affecting the autonomic nervous system can negate the changes in blood vessel compliance, resulting in orthostatic hypotension [9] and an increased risk of falls.

Hypertension is a common condition and use of antihypertensive and vasoactive medications have been associated with falls in older adults, partly because their use is associated with orthostatic changes in addition to adverse effects such as dizziness and gait disturbance [10, 11]. For this reason, more modest target blood pressures of 150/90 mmHg have been set in older adults over 80 years as this group receive marked cardiovascular benefits even at this level of control [12, 13]. This highlights the importance of a medication review by the responsible clinician in both the primary and secondary prevention of falls in older adults [14], particularly if the blood pressure is much lower than the advised target. In addition to having any physical injuries managed, patients need to be assessed for any potential contributing factors to the fall. Interventions may include a reduction in antihypertensive medication to try to prevent a second fall.

This study investigates how antihypertensive medications are managed post-inpatient fall in a high-risk cohort of older adults (≥80 years old) with normal/low blood pressure (< 140 mmHg systolic).

## Methods

### Setting

This work was conducted in a large acute NHS Hospital in the United Kingdom (UK). The hospital has an electronic prescribing and medications administration (EPMA) system known as PICS (Prescribing, Information and Communication System). The system is in use across both inpatient medical and surgical specialties. One benefit of the system is that it also captures data on reported falls across all (~ 1200) inpatient beds [15]. Information from the system is then exported to an audit database on a weekly basis, thus facilitating subsequent analysis.

### Data collection

Data on all reported falls occurring between 1 January and 31 August 2014 were captured from the PICS audit database. For each patient with a documented fall, the first fall per patient was included in the analyses, to ensure that all cases were independent. Falls that occurred within 24 h of admission were excluded, as these cases may have given insufficient time for a patient’s pre-fall medication regimen to be established and reflected on the system. Older adults (≥80 years old) with relatively normal/low blood pressures (< 140 mmHg systolic) were then selected as these patients were considered to be at high risk of further falls, and would therefore require an urgent and thorough medication review as part of a post-fall assessment. Only patients that were prescribed antihypertensive medications as defined by the National Institute for Health and Care Excellence (NICE) guidance [13] and/or other medications with antihypertensive properties (angiotensin converting enzyme inhibitors or angiotensin II receptor blockers (ACEi/ARB), beta blockers, calcium channel blockers (CCB), diuretics, nitrates and others), were included in the study as shown in Table 1. Information was collected on the total number of antihypertensives prescribed before the fall, in the 24 h after the fall and at discharge. Information was then collected about the changes to the prescription of these individual classes of drugs in the 24 h after the fall and again at discharge including whether they were stopped or started or whether the doses were increased or reduced at these time points.

**Table 1:** Medications with antihypertensive properties captured on PICS for each inpatient fall

ACEi/ARB	Beta blockers	CCB
Enalapril	Atenolol	Amlodipine
Irbesartan	Bisoprolol	Diltaizem
Lisinopril	Carvedilol	Lercanidipine
Losartan	Metoprolol	Nifedipine
Perindopril	Nebivolol	Nimodipine
Quinapril	Propranolol	Tildiem
Ramipril	Sotalol	Verapamil
Telmisartan	Timolol	
Valsartan		
Diuretics	Nitrates	Others
Acetazolamide	Glyceryl trinitrate	Alfuzosin
Bendroflumethiazide	Isosorbide dinitrate	Doxazosin
Bumetanide	Isosorbide mononitrate	Eplerenone
Co-amilofruse		Moxonidine
Furosemide		Prazosin
Indapamide		Spironolactone

**Fig. 1:**
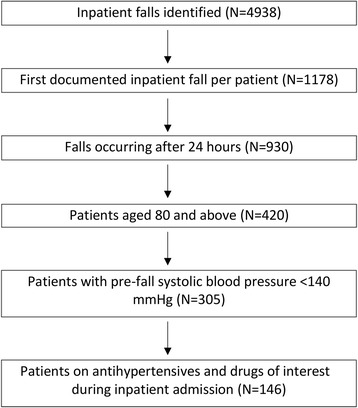
Inclusion and exclusion criteria for inpatient falls

The study flowchart is reported in Fig. 1.

For the patients included in the study, a range of demographic and physiological factors were captured for the pre-fall stage, defined as the period of time between 24 h after admission and the fall being documented. For multiple data entries, data closest to the point of the fall occurring was captured. All active prescriptions for the patients were recorded at this time, as well as post-fall (within 24 h after the fall) and at discharge (those documented on the discharge prescription).

### Statistical methods

Initially, the data were analysed on a patient level. The total number of antihypertensives prescribed for a patient at the post-fall and discharge time points were compared to pre-fall using Wilcoxon’s signed-rank tests.

The data were then analysed on a medication level to consider dose changes and differences in prescribing behaviour across classes of medications. Where patients were prescribed a medication, the total daily dose was calculated at the three time points, with a dose of zero used if the medication was not being prescribed at that time. Wilcoxon’s signed-rank tests were then used to assess the change over time in these doses. The changes in doses were then compared between the medications. To account for the differing magnitudes of doses across the medications, an ordinal variable was produced, which categorised the change in dose as: stopped, reduced, no change, increased or started. This was then compared between the medications using a Kruskal-Wallis test.

All analyses were performed using IBM SPSS 22 (IBM Corp. Armonk, NY), with *p* < 0.05 deemed to be indicative of statistical significance throughout. Continuous variables are reported as medians and interquartile ranges (IQRs).

## Results

Data were available for 146 patients (Table 2). The median pre-fall systolic blood pressure was low across the entire cohort, 122 mmHg (IQR: 109-127 mmHg). Most patients in the study were under the care of the medical directorate and the median time to fall in the cohort was 7.2 days from admission.

**Table 2:** Overall Patient Demographics

Age at Admission [Years]	86 [83–90]
Gender	
Male	79 [54%]
Female	67 [46%]
Ethnicity	
Caucasian	135 [92%]
Other	11 [8%]
Directorate^*a*^	
Medical	111 [77%]
Surgical	34 [23%]
Days from Admission to Fall	7.2 [2.7–16.5]
Pre-Fall Systolic BP	122 [109–127]
Pre-Fall Diastolic BP	64 [57–70]
Pre-Fall Heart Rate	79 [65–87]

Data reported as median [IQR] or N [%] as applicable

^a^Based on *N* = 145, after excluding one Critical Care patient

In the pre-fall period, most patients were prescribed one (*n* = 66, 45%) or two (*n* = 35, 24%) antihypertensives (Table 3). In the 24 h after the fall 120 patients (*n* = 120, 82%) were taking the same number of antihypertensive medications as they were before; only 19 patients (13%) had a reduction in the number of medications, whilst the number of medications increased in seven patients (5%) during that period.

**Table 3:** Number of antihypertensives prescribed

	Pre-Fall	Post-Fall	Discharge
No. Antihypertensives			
0	9 [6%]	13 [9%]	33 [23%]
1	66 [45%]	66 [45%]	55 [38%]
2	35 [24%]	33 [23%]	28 [19%]
3	20 [14%]	18 [12%]	17 [12%]
4	10 [7%]	11 [8%]	7 [5%]
5	4 [3%]	3 [2%]	5 [3%]
6	2 [1%]	2 [1%]	0 [0%]
7	0 [0%]	0 [0%]	1 [1%]
Change from Pre-fall		*P* = 0.031*	*P* < 0.001*
Fewer	–	19 [13%]	51 [35%]
Same Number	–	120 [82%]	73 [50%]
More	–	7 [5%]	22 [15%]

*Wilcoxon’s signed-rank test comparing the number of hypertensives with the pre-fall period

Analyses were then performed to consider whether changes were made in the post-fall period to the doses of the antihypertensive agents prescribed. Compared to the pre-fall period, no significant change in doses was detected post-fall (*p* = 0.129, Table 4), with 86% of prescriptions remaining the same. Only 9% (*n* = 26) of the antihypertensive classes assessed were either stopped or reduced immediately post-fall. In addition, 11 new antihypertensives were prescribed at this time (Table 4).

**Table 4:** Changes to prescriptions from pre-fall to 24 h post-fall

		Change Pre-Fall to Post-Fall				
	N^a^	Stopped	Reduced	No Change	Increased	Started
ACEi/ARB	52	6 [12%]	0 [0%]	44 [85%]	0 [0%]	2 [4%]
Beta blockers	53	4 [8%]	0 [0%]	46 [87%]	0 [0%]	3 [6%]
CCBs	26	2 [8%]	0 [0%]	24 [92%]	0 [0%]	0 [0%]
Diuretics	85	9 [11%]	3 [4%]	67 [79%]	1 [1%]	5 [6%]
Nitrates	41	0 [0%]	1 [2%]	39 [95%]	0 [0%]	1 [2%]
Other	22	1 [5%]	0 [0%]	21 [95%]	0 [0%]	0 [0%]
Total	279	22 [8%]	4 [1%]	241 [86%]	1 [0%]	11 [4%]

Comparison of pre-fall vs. post-fall doses across all drug groups (Wilcoxon’s signed-rank test): *p* = 0.129

^a^The number of cases where the medication was prescribed in either the pre-fall or post-fall period

At discharge, half of the patients (*n* = 73, 50%) remained on the same number of antihypertensive medication as on admission, just over a third of patients (*n* = 51, 34%) were on a fewer number and 15% (*n* = 22) were on more (Table 3). However, between the pre-fall and discharge period, a significant reduction in doses of antihypertensives was detected (*p* < 0.001, Table 5), with 7% of prescriptions changed to lower doses, and a further 27% stopped completely. Nonetheless, 4% of prescriptions were changed to a higher dose and 12% of new prescriptions for antihypertensives were started by the time of discharge. Across the individual antihypertensive classes a significant difference was detected across the groups (*p* = 0.022), with the ACEi/ARB group found to have the most frequent dose reductions, with 9% of prescriptions being reduced in dose, and 42% stopped completely by discharge.

**Table 5:** Changes to prescriptions from pre-fall to discharge

		Change Pre-Fall to Discharge				
	N^a^	Stopped	Reduced	No Change	Increased	Started
ACEi/ARB	53	22 [42%]	5 [9%]	22 [42%]	1 [2%]	3 [6%]
Beta blockers	59	9 [15%]	3 [5%]	35 [59%]	3 [5%]	9 [15%]
CCBs	31	9 [29%]	0 [0%]	16 [52%]	1 [3%]	5 [16%]
Diuretics	92	28 [30%]	7 [8%]	38 [41%]	7 [8%]	12 [13%]
Nitrates	46	7 [15%]	5 [11%]	28 [61%]	0 [0%]	6 [13%]
Other	25	9 [36%]	1 [4%]	11 [44%]	1 [4%]	3 [12%]
Total	306	84 [27%]	21 [7%]	150 [49%]	13 [4%]	38 [12%]

^a^The number of cases where the medication was prescribed either pre-fall or at discharge

Comparison of pre-fall vs. discharge doses across all drug groups (Wilcoxon’s signed-rank test): *p* < 0.001 Comparison of the change in doses from pre-fall to discharge between drug groups (Kruskal-Wallis test): *p* = 0.022

## Discussion

This retrospective study conducted in a large acute NHS hospital explored how antihypertensive medications are managed post-inpatient fall. Older adults (≥80 years old) with relatively low blood pressures (< 140 mmHg systolic) were included in the study, as they were potentially at high-risk of further falls. It was assumed that any changes to antihypertensive medications reflected “a medication review”. In some cases, a review may have been conducted and a decision made not to change the medication. As such, the rates of prescription review could not be implied.

The study demonstrated that few patients had their antihypertensives altered immediately post-fall, with most patients remaining on the same number of medications and at the same doses. This was of some concern, particularly since this was a cohort of patients with relatively low pre-fall blood pressures. Whilst the cause for this was unknown, it should have warranted a close review of patients’ antihypertensive medications with a view to reducing or stopping doses, particularly once a fall had occurred. This alteration occurred in only a small number of cases. This may reflect a lack of awareness of guidelines on the management and prevention of falls, which emphasises the importance of reviewing at risk medications as part of a multifactorial risk assessment for all patients, particularly those aged over 65 years [3]. The UK Single Competency Framework for all prescribers also highlights the need for management plans to be adapted as a result of patient monitoring, comorbidities and preferences [16]. Reassuringly, where prescriptions were changed, the tendency was to reduce the numbers and doses of antihypertensives prescribed. However, in a small number of cases, new antihypertensive medications were started in the 24 h following the fall.

At the point of discharge, changes in antihypertensive prescribing were observed more frequently, although half of the cohort was still on the same number of medications at this point and almost half (49%) of the prescriptions had no change in dose. Reductions in the numbers and/or doses of antihypertensives, relative to pre-fall, were only observed in approximately one third of the cohort at discharge.

All antihypertensives assessed in the study had the propensity to cause vasodilator effects and thus increase the risk of falls. According to the screening tool of older person’s prescriptions (STOPP) criteria, such antihypertensive medications should only be stopped in patients with persistent postural hypotension [17]. As such, it may have been that only some patients had documented postural changes and, thus, few had their antihypertensive medication(s) either stopped or reduced. Whilst the study documented pre-fall blood pressures, it was not possible to determine if postural changes were the cause as standing blood pressures were not documented.

In regard to doses of antihypertensives, whilst no significant difference was seen between the pre-fall and post-fall period, there was a significant change in medication doses between the pre-fall and discharge period, with around a third of antihypertensives either stopped or reduced. The more frequent change in doses at discharge could reflect the input of pharmacists who tend to review medications prior to patients being sent home. Interestingly however, some patients were prescribed a greater number of antihypertensives at both the post-fall and discharge period which might be of concern if it caused a lower blood pressure and thereby increased the risk of further falls.

The most significant changes to antihypertensive doses in the discharge period occurred in the ACEi/ARB group. This may reflect adherence to national guidelines, where this group of medications are not considered first-line in older patients with hypertension. In addition, it may be that medical professionals feel that it is more critical to alter doses of these medications compared to other medications analysed in the study. Indeed, stopping ACEi or ARBs is common place in patients with suspected renal dysfunction, which occurs more commonly in older patients.

### Limitations

This study was based on falls data recorded within PICS, which is reliant on accurate documentation. Medication data recorded in PICS allowed us to determine changes that occurred across three time points accurately. However, the reasons for any changes, or lack of changes to regimens were not documented. As such, any changes to post-fall or discharge medications were assumed to be related to the inpatient fall event. In addition, for patients without documented prescription changes, it is unknown if the prescriptions were either not reviewed or whether an active decision was made not to change prescriptions following review. Finally, the reasons for falls occurring could not be determined in the study due to incomplete data.

## Conclusions

Whilst there was evidence that some patients had their antihypertensive medications reviewed and altered at discharge, most patients had no documented changes to their antihypertensive medications immediately post-fall, which suggests that a review did not take place. This suggests that patients’ medications may not be adequately reviewed as part of the multi-factorial assessment of each fall, as recommended by national guidance, and broadly by prescribing competencies. This emphasises the need to raise awareness of the guidelines and provide education about medications associated with falls, how best to adjust regimens and monitor effects in patients, to improve standards of care.

## Abbreviations

ACEi/ARB: Angiotensin converting enzyme inhibitors and angiotensin II receptor blockers
CCB: Calcium channel blockers
EPMA: Electronic Prescribing and Medications Administration
IQR: Interquartile range
NHS: National Health Service
NICE: National Institute for Health and Care Excellence
PICS: Prescribing, Information and Communication System
STOPP: Screening Tool of Older Person’s Prescriptions
UK: United Kingdom
